# Pathways to the emergency department - a national, cross-sectional study in Sweden

**DOI:** 10.1186/s12873-022-00619-3

**Published:** 2022-04-07

**Authors:** Joakim Henricson, Ulf Ekelund, Jens Hartman, Bruno Ziegler, Lisa Kurland, Daniel Björk Wilhelms

**Affiliations:** 1 Department of Biomedical and Clinical Sciences, Faculty of Health Sciences, Linköping University, Linköping, Sweden; 2 Department of Emergency Medicine, Local Health Care Services in Central Östergötland, Region Östergötland, SE-58185 Linköping, Sweden; 3 Department of Emergency Medicine, Skåne University Hospital, Lund University, Lund, Sweden; 4 Department of Emergency Medicine Örebro University Hospital, Örebro University, Örebro, Sweden; 5 Division of Drug Research, Department of Biomedical and Clinical Sciences, Faculty of Health Sciences, Linköping University, Linköping, Sweden

**Keywords:** Sweden, Triage, Emergency service, Hospital, Demography, After hours care, Delivery of health care

## Abstract

**Background:**

Swedish Emergency Departments (EDs) see 2.6 million visits annually. Sweden has a strong tradition of health care databases, but information on patients’ pathways to the ED is not documented in any registry.

The aim of this study was to provide a national overview of pathways, degree of medical acuteness according to triage, chief complaints, and hospital admission rates for adult patients (≥18 years) visiting Swedish EDs during 24 h.

**Methods:**

A national cross-sectional study including all patients at 43 of Sweden’s 72 EDs during 24 h on April 25th, 2018. Pathway to the ED, medical acuteness at triage, admission and basic demographics were registered by dedicated assessors present at every ED for the duration of the study. Descriptive data are reported.

**Results:**

A total of 3875 adult patients (median age 59; range 18 to 107; 50% men) were included in the study. Complete data for pathway to the ED was reported for 3693 patients (98%). The most common pathway was self-referred walk-in (*n* = 1310; 34%), followed by ambulance (*n* = 920; 24%), referral from a general practitioner (*n* = 497; 1 3%), and telephone referral by the national medical helpline “1177” (*n* = 409; 10%). In patients 18 to 64 years, self-referred walk-in was most common, whereas transport by ambulance dominated in patients > 64 years.

Of the 3365 patients who received a medical acuteness level at triage, 4% were classified as Red (Immediate), 18% as Orange (very urgent), 47% as Yellow (Urgent), 26% as Green (Standard), and 5% as Blue (Non-Urgent).

Abdominal or chest pain were the most common chief complaints representing approximately 1/3 of all presentations.

Overall, the admission rate was 27%. Arrival by ambulance was associated with the highest rate of admission (53%), whereas walk-in patients and telephone referrals were less often admitted.

**Conclusion:**

Self-referred walk-in was the overall most common pathway followed by ambulance. Patients arriving by ambulance were often elderly, critically ill and often admitted to in-patient care, whereas arrival by self-referred walk-in was more common in younger patients.

**Supplementary Information:**

The online version contains supplementary material available at 10.1186/s12873-022-00619-3.

## Background

All 10 million citizens in Sweden have unrestricted access to tax subsidized, public urgent and emergency care, which is provided either by general practitioners (GPs) at primary healthcare facilities, or in hospital-bound emergency departments (EDs).

Swedish EDs have approximately 2.6 million visits annually and the number is steadily increasing [1]. However, the reasons for the increasing ED demand are largely unknown.

Unified national reporting of basic data regarding the emergency department patient population in the National Patient Registry [2] has only recently become mandatory [1]. Surprisingly, however, several basic variables, such as previous healthcare contacts, chief complaints, triage level or mode of arrival to the EDs are not reported to the National Registry.

Patients can gain access to urgent and emergency care through two conceptual pathways. In an emergency the first point of contact is via the national emergency telephone service, 112, which manages pre-hospital ambulance resources. When an acute, but non critical, need arises, the early point of contact is the national medical helpline, 1177, which shares many similarities with the NHS 111 urgent care telephone service [3]. Patients may be directed to either self-care, primary care or to visit the ED. Availability to primary healthcare facilities and on-call GPs is generally limited to office hours on weekdays. The medical help line is advisory and patients with less urgent complaints who are unwilling to wait until office hours for a primary care visit may opt to seek care in an ED.

A retrospective study from 2014 indicated that approximately 16% of all cases processed by the Swedish national emergency telephone service were not in need of ambulance services and could have been adequately managed in primary healthcare [4], and is comparable to most other industrialized countries [5]. There is an ongoing debate in Sweden on the appropriate use of ED care. In Sweden, this debate has come to specifically focus on self-referred walk-in patients and low-acuity patients being recommended to visit the ED after contact with the national medical helpline or their designated primary healthcare facility [6].

Reviews based on large international materials indicate that 20 to 40% of attendances at EDs across the world are inappropriate in the sense that the specific diagnostic and therapeutic resources of a hospital-bound ED are not needed for management of these patients [7, 8]. Many attempts have been made to help identify inappropriate ED attendances or cases which may be more suitable to manage in primary healthcare [7, 8], and several approaches have been suggested [7–9]. Interestingly, relatively simple signals, such as mode of arrival to the ED, seem to be strongly indicative of prognosis, with a reported seven times higher crude mortality rate for patients arriving by ambulance as compared to all other modes of arrival in a 2017 study from England [10]. So far, however, the predictive value of the mode of arrival to the ED for other common outcomes, such as the need for hospital admission, has not been widely investigated and, in contrast to the UK or Australia [11, 12], comprehensive national data on patients’ arrival pathways to EDs in Sweden is lacking.

Previous single-centre cross-sectional studies on patients’ pathways to EDs in Sweden have indicated that a large proportion of patients arrive to the ED as a result of either formal referrals or informal recommendations resulting from preceding healthcare encounters [6, 13]. Arrival by ambulance has been shown to be the most common mode of arrival, whereas a recommendation to go to the ED provided by the medical helpline 1177 was the second most common pathway leading patients to the ED. [6]

Without nation-wide data on pathways leading patients to the ED in relation to age, level of medical acuteness, chief complaints, admission rates and other basic information, the debate on increasing ED visits and unnecessary emergency care will, however, remain largely un-informed.

Therefore, the aim of this study was to determine that such data can indeed be collected at a national level in Swedish EDs, and to provide an overview of this basic information for patients visiting Swedish EDs during 24 h.

## Methods

This was a national, cross-sectional study including all adult patients (18 years or above) attending Swedish EDs during 24 h on April 25th, 2018.

The study was approved by the regional ethics review board in Linköping (permit number 2018/50–31) and performed in accordance with the Declaration of Helsinki and with relevant guidelines and regulations. Patients were informed about the study by posters in the waiting rooms at all participating EDs, and consent was presumed unless patients actively declined participation.

Participating sites were recruited in a two-step process: All Swedish EDs were initially contacted by an e-mail to the head of the department, followed by a telephone call. Participation was confirmed in writing by the head of department. Each participating ED was instructed to appoint a local study coordinator who was responsible for the study at the site.

### Acuity assessment

In the present study the reported triage priority according to the triage tool RETTS was used as proxy to indicate medical acuity [14]. RETTS is a five-level triage system developed at the ED at Sahlgrenska University Hospital, Gothenburg, Sweden, in 2005 and relatively uniformly implemented in the Swedish emergency medical services since 2010. RETTS consists of annually updated flowcharts of the most common ED presentations. Each flowchart comprises several emergency signs and symptoms (ESS) which, in combination with assessments of vital signs (respiratory rate, oxygenation, heart rate, blood pressure, reaction level scale [15] or Glasgow coma scale [16] and body temperature) yield a triage colour reflecting the urgency and acceptable waiting time to assessment by a doctor. Blue (non-urgent) indicates a limited need of emergency care, Green a standard need, Yellow an urgent, Orange a very urgent and red an immediate need of emergency care. Triage priority is typically assigned by a nurse or a doctor at, or shortly after, the patient’s arrival at the ED. The priority level obtained using RETTS, is closely related to in-hospital mortality and hospital length of stay [17] and can serve as a proxy for “medical acuity” [18].

### Definitions

Pathway was defined as the immediately preceding healthcare encounter or initiative (in the case of self-referred walk-in patients) to the index ED visit. In some instances, “mode of arrival” has been used in a synonymous fashion, especially in relation to ambulance arrivals.

Self-referred patients without any documented preceding healthcare contacts before presentation to the ED were registered as self-referred walk-in arrivals. Other patients were assigned one of the following categories: national medical helpline 1177; general practitioner with referral; general practitioner without referral; other in-hospital doctor with referral; other in-hospital doctor without referral; internet medical service with referral; internet medical service without referral; referred by other healthcare provider; scheduled return visit; arrival by ambulance service. Only one alternative was allowed for each patient. Patients arriving by ambulance were not asked about previous healthcare encounters in response to a perceived medical emergency, as the use of ambulances for non-urgent medical transports is limited. Instead, there is a separate, taxi-like transport system to cater for non-urgent transport of patients to or between healthcare facilities should the patients be unfit to arrange his or her own transportation. This system also has the capacity to transport bed-bound patients.

### Data collection

Data collection was divided into an initial phase and a secondary phase.

In the initial phase, each ED filled in a form (Additional file 1) to register the pathway leading each adult patient to the ED during the 24-h study period. Typically, this initial registration and checking for formal referrals in the electronic health record systems was performed by dedicated personnel at the front desk. Patients who arrived by ambulance were registered as “arrivals by ambulance” by the receiving ED nurse.

Data collection was supervised by designated local coordinators based on instructions from the central study coordinator. Standardized training was provided in video-format and supplemented by written instructions. The study coordinator and other members of the study team were available to answer any questions or provide assistance in the weeks leading up to and throughout the 24 h study period.

In the second phase of the study, supplemental data on all registered patients were acquired from electronic health records by the local coordinators at each participating ED.

All data was compiled in a custom spreadsheet provided to the local coordinator at each ED and delivered to the central study coordinator. The patients’ identities were not known to the central study team but can be determined in the local records at each participating site.

### Data analysis

The number of patients arriving to the ED by each pathway was summarized and divided by the total number of patients to gain the percentage distribution. A similar procedure was used to calculate percentage distribution of different pathways for each ED that participated in the study.

The total number of patients per reported priority level was calculated and divided by the total number of patients, as well as for each ED respectively, and presented as a percentage distribution. For the assessment of medical acuity, patients classified as Orange and Red according to RETTS were considered as one group named Critical illness.

The distribution of hospital admission rates for each pathway was calculated by adding up all admitted patients per pathway and dividing that by the total number for pathway, respectively.

## Results

**Fig. 1:**
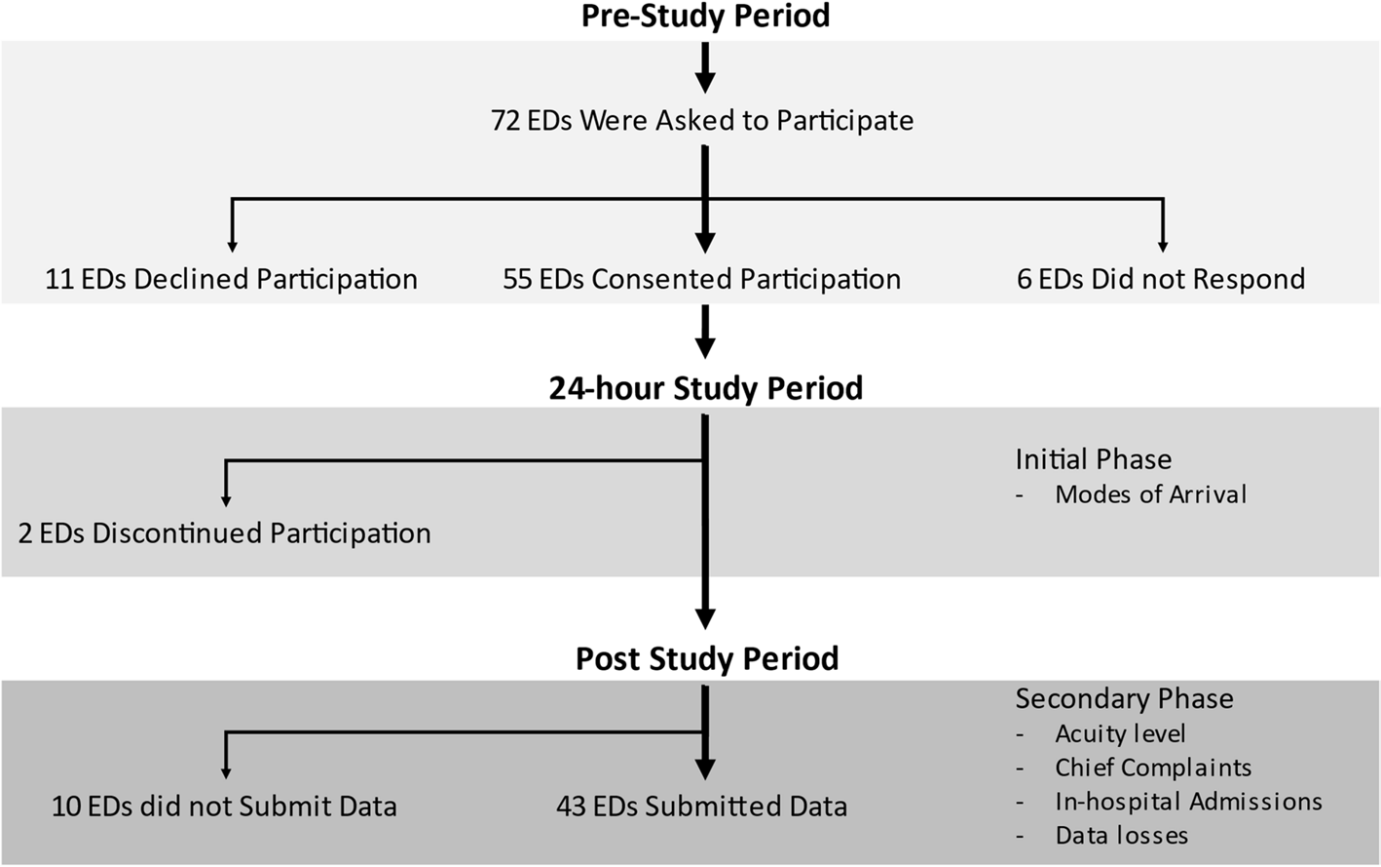
Schematic of the recruitment process, data acquisition processes and number of participating emergency departments (EDs) in the study

Of the invited 72 EDs, 55 agreed to participate, 11 declined and six never responded. Two EDs left the study before submitting data (Fig. 1). Forty-three of the remaining EDs (60%) submitted data for analysis, see Additional file 2. In total, 3875 adult patients were included, median 59 years of age; range 18 to 107 years; 50% men. Completeness of data for the different variables is presented in Table 1.

**Table 1:** Number of complete and missing data

	Mode of Arrival	Sex	Age	Triage Category According to RETTS^a^	Initial Diagnosis	Diagnosis According to RETTS	Admission
**Complete Data**	3693	3782	3785	3277	3776	3640	3718
**Missing Data**	92 (3%)	3 (0.08%)	0	508 (16%)	9 (0.2%)	145 (4%)	67 (2%)

^a^Rapid Emergency Triage and Treatment System (RETTS)

**Fig. 2:**
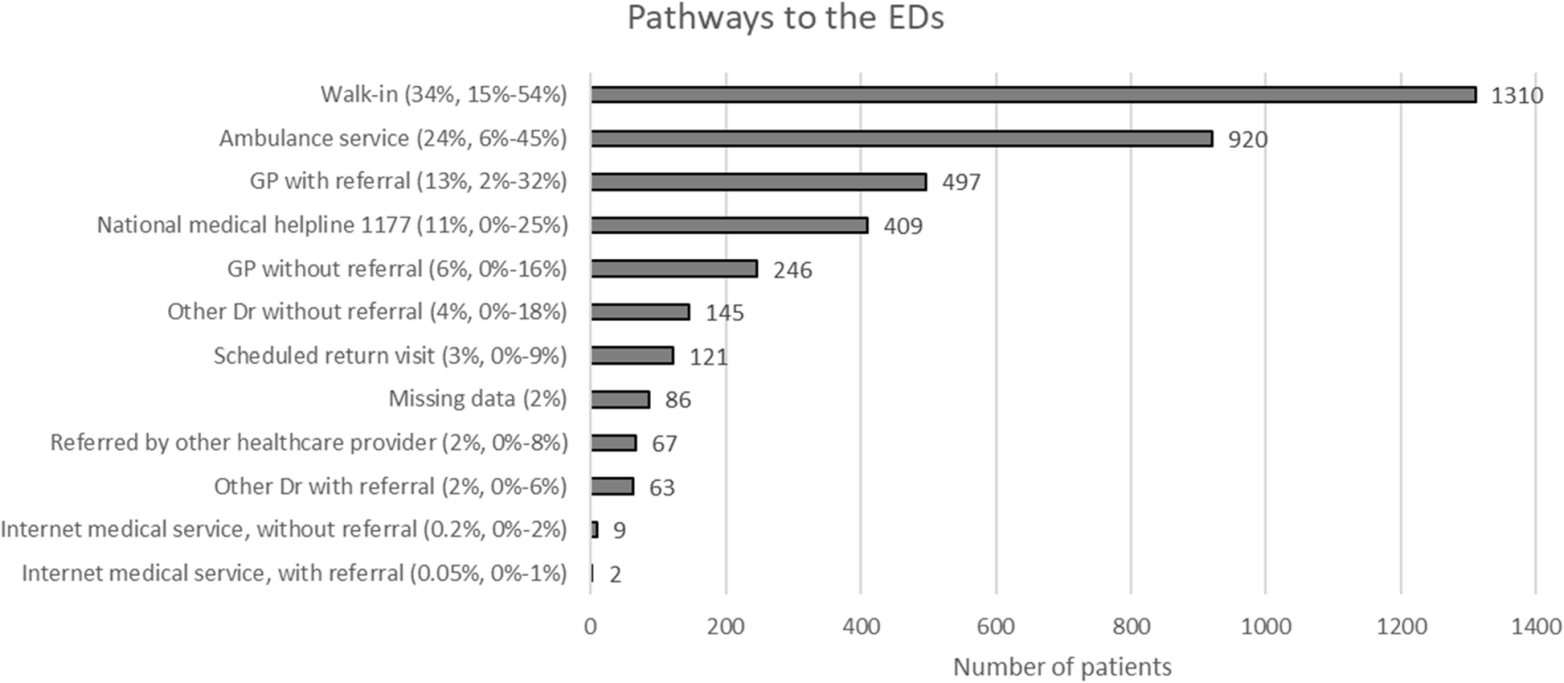
The number and percentage of patients and the pathways among the 3875 patients at the 43 participating emergency departments (EDs) during the 24-h study period. The percental range (%) show the the lowest and highest reported percentage for each mode of arrival among the 43 participating EDs

Figure 2 shows that the most common pathway leading to the ED was self-referred walk-in (34%), followed by arrival by ambulance (24%), referral from a general practitioner (13%), and referral from the national medical helpline 1177 (11%).

**Fig. 3:**
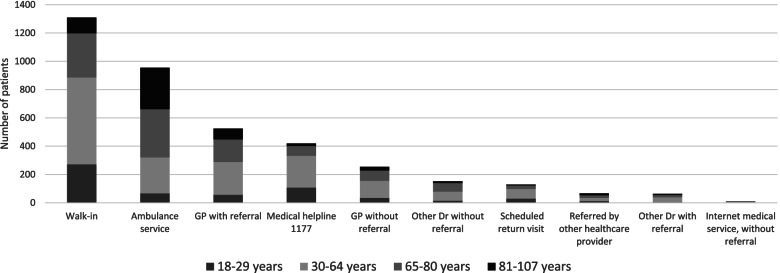
Number of patients per pathway depending on age interval

Self-referred Walk-in was also the most common pathway for patients aged 18 to 29 years (271 of 596; 45%) and 30 to 64 years (614 of 1638; 37%). Arrival by ambulance was the most common mode of arrival for patients between 65 and 80 years (341 of 1081; 32%) and for those 81 years and older (294 of 569; 52%) (Fig. 3).

Of the 3875 included patients, 3365 (87%) were triaged according to RETTS. The remaining patients were either incorrectly triaged using triage levels not included in the RETTS system or lacked triage level). Of all patients receiving a RETTS triage priority, 4% were triaged as Red, 18% as Orange, 47% as Yellow, 26% as Green and 5% as Blue.

Three hundred eighty six of the 920 patients arriving by ambulance services (42%) were triaged as critically ill (RETTS Red or Orange), and 180 of the 1310 self-referred walk-in patients (14%) (Table 2).

**Table 2:** Acuity measured as triage priority according to Rapid Emergency Triage and Treatment System (RETTS) by pathway, as reported by the 43 EDs

	Priority/Acuity According to RETTS						
Pathway	Red	Orange	Yellow	Green	Blue	Missing	Total
Ambulance service	**103** 11 71%	**283** 31% 46%	**349** 38 22%	**126** 14 14%	**3** 0% 2%	**56** 6 11%	**920,**100%
Walk-in	**21** 2 14%	**159** 12% 26%	**509** 39 32%	**328** 25 37%	**83** 6 20%	**210** 16 42%	**1310** 100%
GP with referral	**8** 2% 6%	**58** 12% 10%	**254** 51 16%	**120** 24 14%	**18** 4% 4%	**39** 8% 8%	**497,**100%
Medical Helpline 1177	**6** 1% 4%	**43** 11% 7%	**181** 44 11%	**114** 28 13%	**15** 4% 4%	**50** 12 10%	**409,**100%
GP without referral	**4** 2% 3%	**22** 9% 4%	**117** 48 7%	**81** 33 9%	**7** 3% 2%	**15** 6% 3%	**246,**100%
Other Dr. without referral	**2** 1% 1%	**24** 17% 4%	**58** 40 4%	**40** 28 5%	**5** 3% 1%	**16** 11 3%	**145,**100%
Other Dr. with referral	**1** 2% 1%	**9** 14% 1%	**28** 44% 2%	**12** 19 1%	**5** 8% 1%	**8** 3% 2%	**63,**100%
Referred by other healthcare provider	**0** 0% 0%	**8** 12% 1%	**28** 42 2%	**23** 34 3%	**6** 9% 1%	**2** 3% 0%	**67,**100%
Scheduled return visit	**0** 0% 0%	**4** 3% 1%	**48** 40% 3%	**38** 31 4%	**18** 15 4%	**13** 11 3%	**121,**100%
Internet medical service, with referral	**0** 0% 0%	**0** 0% 0%	**2**100 0%	**0** 0% 0%	**0** 0% 0%	**0** 0% 0%	**2**100%
Internet medical service, without referral	**0** 0% 0%	**0** 0% 0%	**2** 22% 0%	**5** 56% 1%	**1** 11% 0%	**1** 11% 0%	**9**100%
Missing						**86,**100% 17%	**86,**100%
**Total**	**145** 100%	**610** 100%	**1576** 100%	**887** 100%	**161** 100%	**496** 100%	**3875** 100% 100%

Bold numbers indicate actual numbers of registered patients for the respective acuity levels and pathway. Horizontal percentages represent distribution of acuity level per pathway. Vertical percentages represent the distribution of pathway per acuity level. *GP* General practitioner. Other Dr. = another in-hospital doctor

Table 3 shows that the most common chief complaint at ED presentation was abdominal pain (524; 14%), followed by chest pain (369; 10%) and breathing problems (278; 7%).

**Table 3:** The 20 most common chief complaints as reported by the patients upon presentation to a ED, categorized in accordance with the triage system (RETTS)

	Average		Range	
Chief Complaints	N	Percent	Highest	Lowest
			Percent	Percent
Abdominal pain/Flank pain	524	14%	26%	0%
Chestpain/Rib cage pain	369	10%	23%	4%
Respiratory disorder/Dyspnea/Respiratory distress	278	7%	15%	0%
Injury hand/Arm	212	5%	22%	0%
Extremity pain	144	4%	11%	0%
Injury/Trauma/Head	129	3%	9%	0%
Vertigo	115	3%	12%	0%
Non-specifik illness	110	3%	18%	0%
Injury Foot	106	3%	9%	0%
Neurological loss/TIA	97	3%	18%	0%
Non-specifik infection	96	2%	10%	0%
Fever	86	2%	7%	0%
Headache	84	2%	7%	0%
Abnormal heart rate	82	2%	9%	0%
Back pain	77	2%	8%	0%
Injury knee/Lower leg	73	2%	10%	0%
Injury hip/Femur	65	2%	9%	0%
Urinary complications	52	1%	7%	0%
Non-specifik extremity complications	48	1%	18%	0%
Sickness/Fatigue	48	1%	27%	0%
Other/Missing	1080	28%		
**Total**	**3875**	**100%**		

The Average column shows the total number and proportion of patients with a specific chief complaint in relation to the total number of patients in the study (3875). The Percent colums, in the Range segment, indicate the higest and lowest percentage of patients with a chief complaint as reported per emergency department (ED) in relation to the total number of patients at each ED

The overall hospital admission rate for the entire cohort was 27% (1056 patients). Arrival by ambulance service was associated with the highest rate of hospital admission at 53% (486 of 920), followed by self-referred walk-in at 17% (222 of 1310), see Table 4. The admission rate for those triaged as critically ill was 59% (446 of 755), and it was 26% (405 of 1574) for the Yellow, 13% (112 of 884) for the Green, and 2% (4 of161) for the Blue patient groups, respectively.

**Table 4:** The number and percentage of patients admitted to in-hospital care per to pathway

	Admission	Total	Percentage
Ambulance service	486	920	53%
Walk-in	222	1310	17%
GP with referral	132	497	27%
Medical Helpline 1177	58	409	14%
GP without referral	44	246	18%
Other Dr. without referral	40	145	28%
Other Dr. with referral	22	63	35%
Referred by other healthcare provider	19	67	28%
Scheduled return visit	14	121	12%
Internet medical service, with referral	1	21	5%
Internet medical service, without referral	0	9	0%
Missing data	18	86	21%

*GP* General practitioner. Other Dr. = another in-hospital doctor

## Discussion

This is the first nationwide study in Sweden providing an overview of the pathways leading patients to the ED along with basic information on age, level of medical acuity, chief complaints and admission rates. In general, the current study shows that this type of data can be acquired in Swedish EDs with a high degree of completeness, although continuous reporting will require automation of the reporting process. This is an important finding, since comprehensive data even on this very basic level is currently lacking in Sweden, whereas countries such as Australia and the UK provide this information in open reports [11, 12]. Achieving a similar level of detail in the national reporting on ED attendances will be an essential step to support the development of emergency medicine in Sweden, especially in relation to benchmarking, quality assurance and the development of relevant quality indicators. National data on chief complaints, although not further explored in this report, will also provide important information to guide the development of national guidelines specific to the emerging field of emergency medicine. The main point of including such data in the current report, however, was primarily to show that it is actually feasible to collect such data on a national level.

The snapshot provided by the current study showed that the most common pathway leading to an ED visit was self-referred walk-in, which corresponded to approximately one third of all ED arrivals. A large proportion of the self-referred walk-in patients were young and had less urgent medical needs, as indicated by an overall low triage level and few patients admitted to in-hospital care (17%). Interestingly an even smaller proportion of those patients who were recommended by the national medical helpline 1177 to go to the ED were admitted (14%), suggesting that the ability of the medical helpline to assess the need for in-hospital care is likely limited. Similar patterns have been reported in previous studies which leads to the question whether some of these patients could be managed elsewhere in the healthcare system, and thereby decrease the strain on the EDs. Low acuity as indicated by triage category, as well as self-referral, have previously been reported as common factors in ED patients who could be managed in primary care [9, 19–21].

The causes for the high number of low acuity patients are largely unknown on a deeper, mechanistic level, but limited availability of primary healthcare may partly explain why so many low acuity patients come to the ED. [20, 22, 23] Since primary healthcare facilities in Sweden mostly operate during office hours on weekdays, and usually requires an appointment, there are many reports in the media claiming that the need for primary healthcare appointments far exceeds the capacity of the primary healthcare system. However, since there are no data on “appointments not being made”, i.e., those instances in which a patient correctly contacts the primary healthcare facility but cannot be offered a timely appointment, it is unclear to which extent the many non-urgent patients coming to the ED is an effect of a failing primary healthcare system, and how much of this phenomenon is attributable to patient-related factors. Indeed, some patients may believe that they will receive better care at the hospital ED or overestimate the urgency of their health problem [24, 25]. Another possible contributing factor is that emergency medicine is under development as a medical specialty in Sweden, and other specialities may sometimes still consider the ED as convenient venue for their semi-urgent outpatients.

In contrast, more than half of the patients who arrived by ambulance were admitted (53%). These patients were often elderly and had the highest prevalence of critical illness according to triage priority, indicating that arrival by ambulance may serve as a proxy for increased risk of severe illness. Although we did not investigate mortality in this study, our findings point in the same direction as a recent study from England, where arrival by ambulance was associated by an approximately seven-fold increase in crude mortality compared to all other modes of arrival to the ED. [10] Thus, the indicative effect of arrival by ambulance, as well as other pathways, on the risk for adverse outcomes should be a relevant topic for future studies.

On a systems level, the current study highlights large differences between hospitals in the contributions of the various patient pathways. This may partly be explained by geographical differences in the organization of emergency and acute care since, in some parts of the country, the ED may be the only healthcare facility available even on some weekdays, and an ambulance may be the only available means of transportation for, e.g., the elderly. However, geography alone cannot explain the large variation of low acuity patients arriving as walk-ins, or those who have been in previous contact with either primary healthcare or the 1177 medical helpline. Rather, our results indicate that the decision-making process before ED referrals or recommendations may differ, and that there is a need for clearer criteria for ED care, which is a common topic for discussion also internationally [7, 9, 26]. However, since Sweden is a geographically large, scarcely populated country with most of its 10 million population concentrated to a handful of metropolitan areas in the southern part of the country, there will always be a need for adaptive solutions to provide adequate emergency care to parts of the population living under very different geographical conditions. This means that an ED presentation which is clearly inappropriate in a large city may be the only feasible way to get healthcare in a remote, rural area. This aspect needs to be considered in future work on the appropriateness of ED care in Sweden.

The findings of the current study underscore the need for continuous reporting of information on patients’ pathways to the ED, as well as other basic information on ED patients on a national level. The fact that many of the ED attendances based on recommendations from the national medical helpline 1177 are for low acuity conditions with limited need for in-hospital care, clearly indicates that the system may partly be responsible for the increasing strain on EDs which, in turn, may negatively affect the outcomes of more severely ill patients [21]. The UK and Australia have, in contrast to Sweden, well-developed systems for continuously reporting mode of arrival and other basic information for all ED patients [12, 27] and we strongly suggest that such a model for national reporting be implemented. Such information would provide a better understanding of ED operations and allow us to optimize resource usage and develop relevant quality indicators for emergency medicine in Sweden.

## Limitations

There are some methodological limitations to this study. The initial triage classification and admission rate are rather blunt instruments for estimating medical acuity but are commonly used. Information about previous health care contacts was reported by patients and manually recorded with no means of quality control. Although two thirds of all EDs in Sweden participated, several EDs in the major cities (3 in Stockholm and 2 in Gothenburg) declined participation. However, the participating EDs are geographically evenly distributed across Sweden and include EDs of all sizes, and we therefore believe that the results are generalizable. It is a limitation that the study results are point estimates from one 24-h period. However, the specific date was chosen to represent a day in the middle of the week and not during a holiday period. Further, the RETTS triage scale, which is widely used at Swedish emergency departments, commonly has some local or regional adaptions, although these adaptions should not affect the reported level of priority.

## Conclusion

Self-referred walk-in, arrival by ambulance and referral from primary care, were the most common pathways leading patients to Swedish EDs. Ambulance arrivals were most common in patients > 64 years of age, and self-referred walk-ins dominated in younger patients. Patients arriving by ambulance had the highest level of acuity and hospital admission rates. A nationwide, systematic registration of ED performance measures is necessary to optimize resource usage and develop relevant quality indicators for emergency medicine in Sweden.

## Supplementary Information

**Additional file 1.**

**Additional file 2.**

## Data Availability

The dataset used and analysed during the current study is available from the corresponding author on reasonable request.

## Abbreviations

ED: Emergency Department
GP: General practitioner
RETTS: Rapid Emergency Triage and Treatment System
