# Supplementary material for: Pathways to the emergency department - a national, cross-sectional study in Sweden
Source: BMC Emerg Med. 2022 Apr 7;22:58. doi: 10.1186/s12873-022-00619-3 (PMC8991881; doi:10.1186/s12873-022-00619-3)
Supplement: Supplementary file 1 — Additional file 1. [file 12873_2022_619_MOESM1_ESM.docx]

Appendix 1.

Deltagande klinik/akutmottagningen i:

| Plats för etikett  Med patientdata |
| --- |

*Hur ankom patienten till akuten?* ***Kryssa endast ett alternativ!***

| **Ankommer med ambulans** |  |
| --- | --- |

**Eller**

| **Eget initiativ utan föregående vårdkontakt** | |  |
| --- | --- | --- |
| **Hänvisad av 1177** | |  |
| **Från vårdcentral** | Med remiss |  |
|  | Hänvisad utan remiss |  |
| **Från klinik på sjukhuset** | Med remiss |  |
|  | Hänvisad utan remiss |  |
| **Från vårdgivare på internet** | Med remiss |  |
|  | Hänvisad utan remiss |  |
| **Hänvisad vid sjukvårdskontakt (t.ex. sjukgymnast)** | |  |
| **Återbesök på akutmottagning** | |  |

Participating Clinic/Emergency Department

| Patient data sticker |
| --- |

*What was the patient’s pathway to the emergency?* **Check only one alternative!**

| **Arrival by ambulance** |  |
| --- | --- |

**Or**

| **Self-referred walk-in without prior healthcare contact** | |  |
| --- | --- | --- |
| **Referred by national medical helpline 1177** | |  |
| **From primary care** | By referral |  |
|  | Without referral |  |
| **From in-hospital doctor** | By referral |  |
|  | Without referral |  |
| **Internet medical service** | By referral |  |
|  | Without referral |  |
| **Referred by other healthcare provider** | |  |
| **Return visit** | |  |
