# Supplementary material for: Pathways to the emergency department - a national, cross-sectional study in Sweden
Source: BMC Emerg Med. 2022 Apr 7;22:58. doi: 10.1186/s12873-022-00619-3 (PMC8991881; doi:10.1186/s12873-022-00619-3)
Supplement: Supplementary file 2 — Additional file 2. [file 12873_2022_619_MOESM2_ESM.docx]

Appendix 2. List of the 43 EDs that participated in the study and sent back data.

1 Arvika
2 Avesta
3 Bollnäs
4 Eksjö
5 Enköping
6 Falun
7 Halmstad
8 Helsingborg
9 Jönköping Ryhov
10 Kalix
11 Karlskoga
12 Karlskrona
13 Karlstad
14 Karolinska Solna
15 Kristianstad
16 Kullbergska Katrineholm
17 Kungälv
18 Linköping
19 Ljungby
20 Lund
21 Malmö
22 Mora
23 Motala
24 Mölndal
25 Norrköping
26 S:t Göran
27 Skövde
28 Sollefteå
29 Sunderby Sjukhus
30 Sundsvall
31 Södersjukhuset
32 Torsby
33 Trelleborg
34 Uppsala Akademiska
35 Varberg
36 Visby
37 Värnamo
38 Västervik
39 Växjö
40 Ystad
41 Ängelholm
42 Örebro
43 Örnsköldsvik
